# Phytochemical characterization and pharmacological evaluation of aerial and root parts of *Dalea pazensis* Rusby [Fabaceae]

**DOI:** 10.3389/fphar.2025.1717359

**Published:** 2026-01-05

**Authors:** Mariana Andrea Peralta, Einy Nallybe Bedoya Aguirre, Melisa Fabiana Negro, Javier Echeverría, Maria Daniela Santi, Maria Gabriela Ortega

**Affiliations:** 1 Farmacognosia, Departamento de Ciencias Farmacéuticas, Facultad de Ciencias Químicas, Universidad Nacional de Córdoba, Córdoba, Argentina; 2 Unidad de Investigación y Desarrollo en Tecnología Farmacéutica (UNITEFA-CONICET), Córdoba, Argentina; 3 Departamento de Ciencias del Ambiente, Facultad de Química y Biología, Universidad de Santiago de Chile, Santiago, Chile; 4 Max Planck Institute for Multidisciplinary Sciences, Goettingen, Germany

**Keywords:** *Dalea pazensis*, prenylflavanoids, essential oil, anti-tyrosinase activity, antifungal bioactivity

## Abstract

**Background:**

The *Dalea* genus [Fabaceae] is rich in bioactive flavonoids and contains underexplored species, such as Dalea pazensis Rusby, with potential for antifungal and dermatological applications.

**Purpose:**

Considering the limited knowledge available on *D. pazensis,* this study aims to expand the current understanding of its chemical and biological potential.

**Material and Methods:**

Sequential extraction of *D. pazensis* roots was performed using solvents of increasing polarity. Additionally, essential oil (EO) was obtained from the aerial parts. Extracts and EO were chemically characterized by ultra-performance liquid chromatography–tandem mass spectrometry (UPLC-MS/MS) and gas chromatography/mass spectrometry (GC-MS), respectively. Antifungal activity was evaluated against azole-sensitive and azole-resistant *Candida albicans* strains, while tyrosinase inhibition was assessed in the different extracts using an *in vitro* enzymatic assay.

**Results and discussion:**

The chloroform extract (CDp) exhibited the most potent antifungal activity (minimum inhibitory concentration, MIC = 125 μg/mL), a relevant value considering that the reference drug fluconazole shows an MIC of 32 μg/mL in this resistant strain, underscoring the extract’s significant activity despite azole resistance. CDp showed significant tyrosinase inhibition (half-maximal inhibitory concentration, IC_50_ = 1.27 μg/mL). UPLC-MS/MS analysis identified (2*S*)-5,7,2′,4′-tetrahydroxy-5′-(1‴,1‴-dimethylallyl)-8-prenylflavanone (compound 1) as the major constituent, previously linked to antifungal activity and efflux pump inhibition. EO analysis revealed β-caryophyllene (41.1%) as the main component, suggesting a distinct chemotype within the species.

**Conclusion:**

This is the first chemical report of the EO and the deepening of prenylflavonoid content in different extracts of *D. pazensis*, highlighting the pharmacological relevance of its dual antifungal and antityrosinase profile, a combination of interest for dermatological formulations targeting fungal infections, hyperpigmentation, or post-infectious dyschromias. The findings underscore this species as a promising source of prenylated flavanones with dual antifungal and anti-tyrosinase activity, as well as bioactive volatiles with antifungal activity. The results support its use in developing natural antifungal therapies, particularly against MDR pathogens.

## 1 Introduction

The genus *Dalea* L. [Fabaceae], native to the Americas, extends from the southwestern United States to central Argentina and Chile. Several species within this genus have been used in traditional medicine for their anti-inflammatory and analgesic properties (Densmore, 2005). Phytochemical investigations have revealed a variety of prenylated flavonoids in *Dalea* species, many of which exhibit biological activities, including antimicrobial, antioxidant, and enzyme inhibitory effects (Peralta et al., 2011; Peralta et al., 2012; Peralta et al., 2014; Peralta et al., 2019; Peralta et al., 2025; Negro et al., 2024; Bedoya Aguirre et al., 2025). Our group has extensively studied *Dalea elegans* Gillies *ex* Hook. and *Dalea boliviana* Britton, isolating multiple flavanones—prenylated, chromene-type, and methoxylated derivatives—and evaluating their pharmacological potential. Importantly, several *Dalea* flavonoids exhibit both antifungal and anti-tyrosinase activities, a dual profile of increasing therapeutic interest, as it aligns with emerging needs for treating multidrug-resistant fungal infections and managing hyperpigmentation disorders with natural agents. This combination of activities is particularly valuable from a pharmacological standpoint, as it supports the development of multifunctional compounds capable of addressing co-occurring dermatological conditions, such as infection-associated inflammation and dysregulated melanogenesis, within a single therapeutic framework.

In a recent report, we analyzed the chemical profiles and biological properties of root extracts with different polarities from *D. elegans*, *D. boliviana*, and *Dalea leporina* (Aiton) Bullock, confirming the relevance of flavonoids such as compound 1 [(2*S*)-5,7,2′,4′-tetrahydroxy-5′-(1‴,1‴-dimethylallyl)-8-prenylflavanone] as a bioactive marker of the genus (Peralta et al., 2025).

Despite progress in characterizing non-volatile metabolites from *Dalea*, studies on essential oils (EOs) from this genus remain scarce. Only a few species—including *Dalea scoparia* A. Gray*, Dalea greggii* A. Gray*, Dalea lumholtzii* B.L. Rob. & Fernald*, Dalea carthagenensis* (Jacq.) J.F. Macbr.*, Dalea formosa* Torr.*, Dalea foliolosa* (A. Gray) Barneby, *Dalea strobilacea* Barneby, and *Dalea mutisii* Kunth—have been analyzed for their volatile fractions (McCaughey and Buehrer, 1961; Lucero et al., 2005; Benites et al., 2016; Villa-Ruano et al., 2017; Muñoz-Acevedo et al., 2019; Gilardoni et al., 2020), revealing terpene-rich profiles with potential aromatic and antimicrobial activity. More recently, an EO obtained from *Dalea bicolor* Humb. & Bonpl. ex Willd. [Fabaceae] has been investigated (Esquivel-Campos et al., 2024), further confirming the chemical diversity and bioactive potential of the volatile constituents of the genus.

Based on the available data, we aimed to contribute to the chemical and biological characterization of other *Dalea* species. In this work, we have extensively analyzed *Dalea pazensis* Rusby. *D. pazensis* is a native Andean shrub that grows between 1,000 and 3,500 m in altitude, characterized by violet flowers and robust yellow taproots. Previously, we reported the isolation of four prenylated flavanones from the benzene root extract of this species, including compound 1, reinforcing its chemotaxonomic significance and biological relevance (Santi et al., 2017). On the other hand, no previous chemical or biological data have been reported for the EO of *D. pazensis*.

Considering the growing pharmacological interest in *Dalea* species, particularly as antifungal and anti-tyrosinase agents, and the limited knowledge of *D. pazensis*, this study aimed to expand current understanding of its chemical and biological potential. To this end, we performed a comprehensive analysis of root extracts obtained by sequential solvent extraction, focusing on the detection and quantification of six characteristic flavanones using ultra-performance liquid chromatography–tandem mass spectrometry (UPLC-MS/MS). In parallel, the EO from the aerial parts of *D. pazensis* was obtained and chemically characterized by gas chromatography/mass spectrometry (GC-MS). The antifungal activity of both the root extracts and the EO was evaluated against *Candida albicans* strains, including an azole-resistant clinical isolate. The extracts were also assessed for their tyrosinase inhibitory capacity. Together, these investigations aim to enhance the phytochemical and bioactive profile of *D. pazensis*, underscoring its potential as a source of antifungal and dermatologically relevant compounds.

## 2 Materials and methods

### 2.1 Chemicals and reagents

Chromatographic-grade acetonitrile (ACN), dimethylsulfoxide (DMSO), and methanol (MeOH) were obtained from Merck (Darmstadt, Germany), while formic acid was acquired from the same supplier. The *n*-hexane was purchased from J.T. Baker (New Jersey, United States). Tyrosinase (EC 1.14.18.1) from mushrooms (8503 U/mg and 6540 U/mg), kojic acid (purity: 99%), and L-tyrosine (purity: 99%) were obtained from Sigma Chemical Co. (St. Louis, MO, United States). Fluconazole (FLZ, purity ≥98%) was purchased from Sigma-Aldrich Co. (St. Louis, MO, United States). Sabouraud dextrose agar (SDA) and Sabouraud glucose broth (SGB) were purchased from Britania S.A. (Buenos Aires, Argentina).

### 2.2 Plant material

*D. pazensis* were located and collected in July 2018 in Yotala, a locality near the city of Sucre, Bolivia (19°08′53″S, 65°15′48″W, 2,543 m above sea level). Specialized personnel from the Botanical Museum of Southern Bolivia identified the plant, and a voucher specimen (961A, Portal E. & López C.D.) was deposited in the same institution. The plant material was shade-dried in a cool, well-ventilated area for 20 days under laboratory conditions. It was periodically stirred to ensure proper aeration of the inner layers. Once dried, all foreign material was removed. The aerial parts of *D. pazensis* were separated from the roots and subsequently ground using a Retsch-Mühle knife mill equipped with a No. 6 sieve for further extractions. The details of plant materials, extracts and chemical characterization were presented as per ConPhyMP guidelines (Heinrich et al., 2022).

### 2.3 Preparation of the vegetal material

#### 2.3.1 Preparation of extracts

**FIGURE 1:**
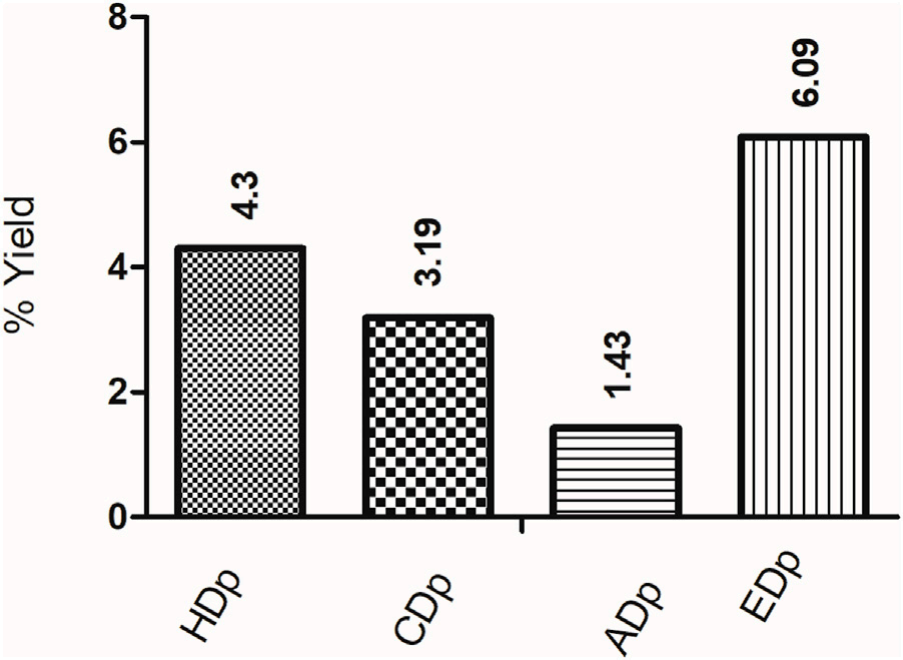
Yields of plant extracts obtained from *D. pazensis* are expressed as percentages based on the weight of dry plant material (grams of extract per 100 g of dry plant material). HDp: *D. pazensis* hexane extract, CDp: *D. pazensis* chloroform extract, ADp: *D. pazensis* ethyl acetate extract, EDp: *D. pazensis* ethanol extract.

A total of 35 g of *D. pazensis* roots was extracted using a Soxhlet apparatus with various solvents in sequence at different temperatures (40 °C–60 °C), depending on the solvent used, for 24 h each. 500 mL of each organic solvent was used, starting with hexane, then chloroform, ethyl acetate, and finally ethanol. The solvent was evaporated to obtain four crude extracts, with yields shown in Figure 1. These dried extracts were labeled as follows: *D*. *pazensis* hexane extract (HDp), *D. pazensis* chloroform extract (CDp), *D. pazensis* ethyl acetate extract (ADp), and *D. pazensis* ethanol extract (EDp).

#### 2.3.2 Essential oil extraction

The extraction of EO from the aerial parts of *D. pazensis* was performed using the method described by Villa-Ruano et al. (2017), using a steam distillation apparatus (Villa-Ruano et al., 2017). Briefly, 300 g of dried aerial plant material was processed in multiple distillations, each consisting of 30 g of plant material per 100 mL of distilled water. Each distillation was carried out for 3 h to ensure complete extraction. The resulting hydrodistillate was collected and extracted with *n*-hexane. Residual water was removed by anhydrous sodium sulfate treatment. The EO was stored at 4 °C in amber glass vials until further analysis. EO yield and density were determined as previously described by Villa-Ruano et al. (2017). The EO yield was calculated as the percentage volume-to-weight (v/w) of oil obtained relative to the initial dry weight of the plant material used for distillation. After each distillation, the EO volume was measured, and the cumulative yield from all batches was expressed as milliliters of EO per 100 g of dry plant material.

### 2.4 Chemical evaluation

#### 2.4.1 Reference compounds: purification and identification

The compounds isolated from *D. elegans* and *D. bolivian*a were obtained from their organic extracts using conventional chromatographic techniques. Structural elucidation was performed through nuclear magnetic resonance (NMR) spectroscopy and mass spectrometry, and identification was achieved by comparison with previously reported data. Table 1 summarizes the reference compounds, their plant sources, and the corresponding references.

**TABLE 1:** Compounds evaluated in root extracts of *Dalea pazensis*, along with their plant sources and corresponding references describing their structural characterization.

Compound	Structure	Plant source	Reference
(*2S*)-5,7,2′,4′-tetrahydroxy-5′-(1‴,1‴-dimethylallyl)-8-prenylflavanone (1)	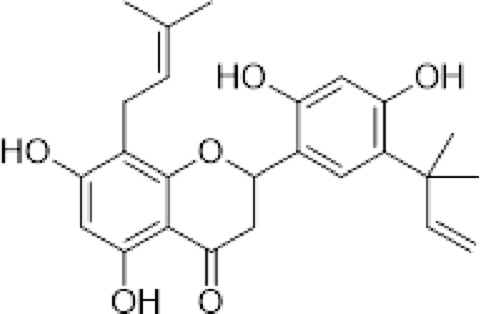	*Dalea elegans* Gillies ex Hook. (roots)	Peralta et al. (2014)
(*2S*)-5,7-dihydroxy-8-prenylflavanone (2)	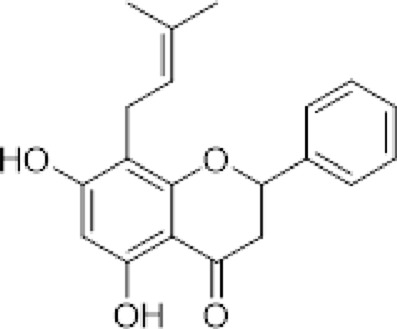	*Dalea elegans* Gillies ex Hook. (aerial parts)	Peralta et al. (2014)
(*2S*)-7-hydroxy-5-methoxy-6-methylflavanone (3)	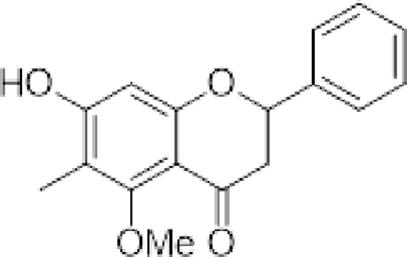	*Dalea elegans* Gillies ex Hook. (aerial parts)	Peralta et al. (2014)
(*2S*)-5,7,2′-trihydroxy-8,3′-diprenylflavanone (4)	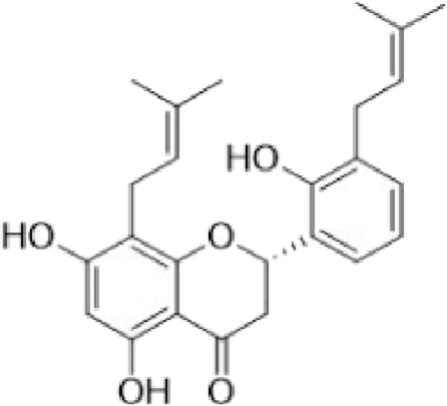	*Dalea boliviana* Britton (roots)	Peralta et al. (2011)
(*2S*)-5,7,2′-trihydroxy-5′-(1‴,1‴-dimethylallyl)-8-prenylflavanone (5)	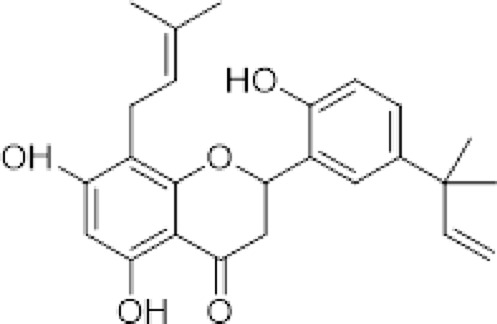	*Dalea boliviana* Britton (roots)	Peralta et al. (2011)
(−) -(*2S*)-5,2′-dihydroxy-6″,6″-dimethylchromeno-(7,8:2″,3″)-flavanone (6)	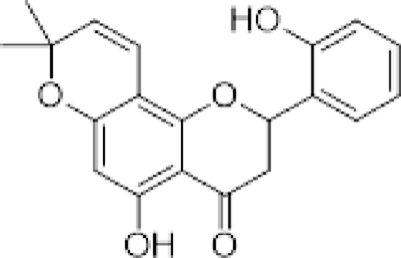	*Dalea boliviana* Britton (roots)	Negro et al. (2024)

#### 2.4.2 UPLC-MS/MS analysis

UPLC/MS-MS experiments were conducted according to the methods described by Gaudio et al. (2020) and Peralta et al. (2025). An Acquity UPLC H-Class, Waters, equipped with a quaternary pump, autosampler, and a triple quadrupole mass spectrometer Xevo TQ-S Micro (Waters, United States) was employed. A Waters BEH C-18 (2.1 × 50 mm, 1.7 μm at 30 °C) was used for the chemical separation using an isocratic method (20% H_2_O, 0.1 v/v formic acid, 80% ACN, and 0.1 v/v formic acid, at a flow of 0.20 mL/min). The different extracts from *D. pazensis* (3 mg/mL in ACN), the reference compounds (1 mg/mL in ACN), and the solvents were filtered through a 0.22 µm Millipore filter, and 5 µL of each was injected in duplicate. Tandem mass spectrometry (MS/MS) analyses were performed in negative-ion mode. Other parameters employed for the study of the reference compounds were: a capillary voltage of 2.4 kV, and desolvation gas (nitrogen) at a flow rate of 650 L/h. The temperatures of the desolvation and source block were 350 °C and 150 °C, respectively. The identification of 1-6 flavonoids was achieved by comparing their retention times and fragmentation patterns with those of the target flavonoids using Multiple Reaction Monitoring (MRM) transitions, as previously reported (Gaudio et al., 2020; Bedoya Aguirre et al., 2025; Peralta et al., 2025). Other relevant information about the reference compounds (precursor, multiple product ions, collision energy, cone voltage, and retention times), as well as the MRM methodology, was included as Supplementary Material (Supplementary Table S1).

The relative content of each compound (1–6) identified in the extracts (Supplementary Table S2) was assigned according to the relative intensities observed by the quantifier transition of each flavonoid (Gaudio et al., 2020; Bedoya Aguirre et al., 2025). The MassLynx software (Version 4.1) was employed for data acquisition, data processing, and conducting device controls.

#### 2.4.3 GC/MS analysis of the essential oil

Chemical analysis of the EO was conducted according to the methodologies described by (Villa-Ruano et al., 2017; Benites et al., 2016), using gas chromatography-mass spectrometry. The compounds were separated using a GC-MS Clarus 560 SQ8 (PerkinElmer, United States) equipped with a DB-5 column (30 m × 250 μm i.d., 0.25 μm film thickness) (Agilent, United States). The injector temperature was set to 250 °C. Injections (1 µL) were performed in the split mode (1/20), and helium was used as the carrier gas at a constant flow of 1 mL/min. The oven temperature program was set to 60 °C (5 min), increasing at 5 °C/min to 240 °C, then holding for 10 min. The GC–MS interface temperature was 200 °C. The electron-impact mode on the mass spectrometer was set to 70 eV, with a mass scan range of 40–300 amu. Retention indices (RI) were calculated from the analysis of the C8–C21 alkane series (Sigma-Aldrich, Buenos Aires, Argentina) under the same chromatographic conditions. Identification of the compounds was based on comparisons of their mass spectra and RI with those from the NIST-08 Mass Spectral Library (US National Institute of Standards and Technology, Gaithersburg, MD, United States). The amount of each compound was expressed as a relative percentage (%) by normalization to peak area. The relative percentage for each peak was calculated as the proportion of the total area of all detected peaks.

### 2.5 Biological assay evaluation

#### 2.5.1 Inhibition of the tyrosinase enzyme

This assay was carried out according to Peralta et al. (2014), with minor modifications (Peralta et al., 2014). Briefly, 0.25 mL of mushroom tyrosinase solution (200 U/mL) with 0.75 mL of sample solution [conformed by Na_3_PO_4_ buffer (pH 6.8) or solution of each extract (ADp and CDp dissolved in DMSO, at a final concentration of 0.1% v/v) diluted to the appropriate concentrations with the mentioned buffer] was assorted and preincubated at 25 °C for 10 min. Finally, 0.50 mL of a 1.7 mM L-tyrosine solution was incorporated. After 20 min of incubation, the measurement at 475 nm was realized. The control consists of the same mixture without the compounds. As a positive control, Kojic acid was used. The tyrosinase activity inhibition percentage was calculated as follows: % inhibition = [(Abs_control_ - Abs_sample_)/Abs_control_] X 100, being Abs_control_ the absorbance of the control, and Abs_sample_ the absorbance of the experimental sample. The results were reported as the mean ± standard error of the mean (SEM) from three independent experiments. The half-maximal inhibitory concentration (IC_50_) values were calculated using Origin 9.1 software on a compatible computer.

#### 2.5.2 Antifungal assessment

##### 2.5.2.1 Strains

Two clinical *Candida albicans* strains were utilized: one susceptible to imidazole antifungals (SCa, 2.76) and another resistant (RCa, 12.99), generously provided by Dr. T. White (University of Washington, Seattle, United States). The resistant strain exhibits overexpression of *CDR1*, *CDR2*, and *MDR1*-like genes, which are associated with multidrug resistance (MDR) mechanisms (White et al., 2002). Both strains were maintained in Sabouraud glucose broth (SGB) for cultivation. For long-term storage, cultures were preserved at −80 °C in 15% glycerol stocks. Before each assay, cells were subcultured from frozen stocks onto Sabouraud dextrose agar (SDA).

##### 2.5.2.2 Evaluation of antifungal activity

The antifungal properties of the extracts and the essential oil were tested against both SCa and RCa strains at varying concentrations (125, 250, 500, 750, and 1,000 μg/mL) following a standardized microdilution method in 96-well plates (CLSI, 2017), with modifications according to Peralta et al., 2012; Peralta et al., 2012). A starting inoculum of 10^3^ colony-forming units per milliliter (CFU/mL) was prepared in liquid culture medium within the microdilution plate.

Extracts were prepared from stock solutions (100 mg/mL in DMSO) and serially diluted in Roswell Park Memorial Institute (RPMI) 1,640 medium supplemented with glutamine, without sodium bicarbonate, and buffered with 0.164 M 3-(N-morpholino)propanesulfonic acid, with pH adjusted to 7 ± 0.1, and containing 0.2% glucose to achieve the desired concentrations in the incubation medium. A solvent control with the same DMSO concentration was included. All plates were incubated at 36 °C for 24 h before absorbance measurement. Absorbance readings at 540 nm were obtained using a MicroQuant Sunrise microplate spectrophotometer (Tecan, Austria).

The minimum inhibitory concentration (MIC) was defined as the lowest extract concentration that reduced the optical density by at least 50% relative to the untreated control, measured at 540 nm using a microplate reader. Data were analyzed with GraphPad Prism version 5.00 (GraphPad Prism 6.0, San Diego, United States), and the standard error of the mean (SEM) was calculated from three independent assays, each performed in duplicate. Results are presented as mean ± SEM.

### 2.6 Statistical analysis

The results obtained from tyrosinase inhibition were statistically analyzed including ANOVA, followed by Tukey’s test for multiple comparisons, which was performed using GraphPad Prism 6.0 software. A *p*-value ≤0.05 was considered statistically significant. For the antifungal assays, statistical significance of the results was determined by two-way ANOVA followed by Bonferroni’s post-hoc test, with *p* ≤ 0.05 indicating statistical significance.

## 3 Results and discussion

### 3.1 Dried extracts performance

*D. pazensis* extracts show variations in their yield profiles. The highest yield was obtained from the ethanol extract (EDp), followed by hexane (HDp), chloroform (CDp), and ethyl acetate (ADp) extracts (Figure 1). The chemical composition of the roots and their different solvent affinities could explain the differences in extract yields. Further research is needed to clarify the underlying reasons for these variations.

We are currently focusing on identifying bioactive compounds that might be present in these extracts to explore their potential uses.

### 3.2 Essential oil yield

The EO yield from the aerial parts of *D. pazensis* was 1.94% w/w, which is significantly higher than the yields reported for other *Dalea* species. For example, *D. formosa* yielded 0.45% (Lucero et al., 2005) and *D. strobilacea* yielded 0.90% (Benites et al., 2016). Likewise, lower EO yields were reported for *D. scoparia* and *D. greggii* (McCaughey and Buehrer, 1961), as well as *D. mutisii*, from which EO extraction from flowers yielded less than 0.25% w/w (Gilardoni et al., 2020). This relatively higher EO yield in *D. pazensis* highlights not only its potential as a useful source of volatile bioactive compounds but also the significance of species-specific and regional factors in determining EO productivity within the *Dalea* genus.

### 3.3 Biological activity studies

Based on the reported biological activities of extracts and constituents from various *Dalea* species (Gaudio et al., 2020; Peralta et al., 2025). We assessed the tyrosinase inhibitory and antifungal activities of four root extracts from *D. pazensis*, obtained through sequential extraction with solvents of increasing polarity (hexane, chloroform, ethyl acetate, and ethanol). Additionally, based on previous studies on the EOs of other *Dalea* species, the EO from the aerial parts of *D. pazensis* was evaluated for its antifungal activity against *C. albicans*. These assessments aimed to broaden the understanding of the chemical diversity and biological potential of this underexplored species.

### 3.4 Tyrosinase inhibitory activity of *Dalea pazensis* root extracts

**FIGURE 2:**
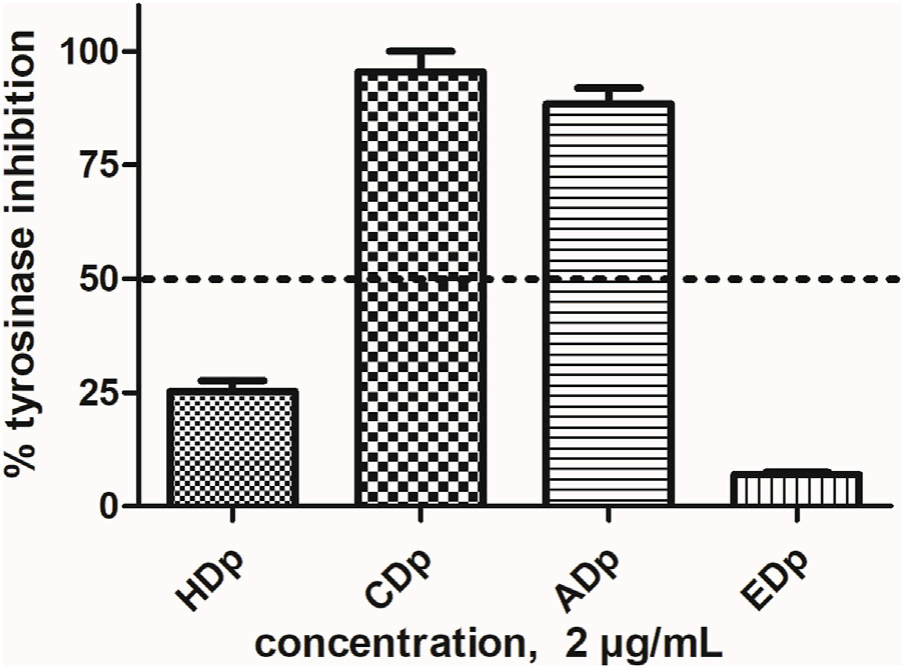
Different extracts from *D. pazensis* as tyrosinase inhibitors at a concentration of 2 μg/mL. Black dotted lines indicate 50% inhibition. HDp: *D. pazensis* hexane extract, CDp: *D. pazensis* chloroform extract, ADp: *D. pazensis* ethyl acetate extract, EDp: *D. pazensis* ethanol extract. Data are presented as mean ± standard error of mean (SEM) from three independent experiments (*n* = 3).

**FIGURE 3:**
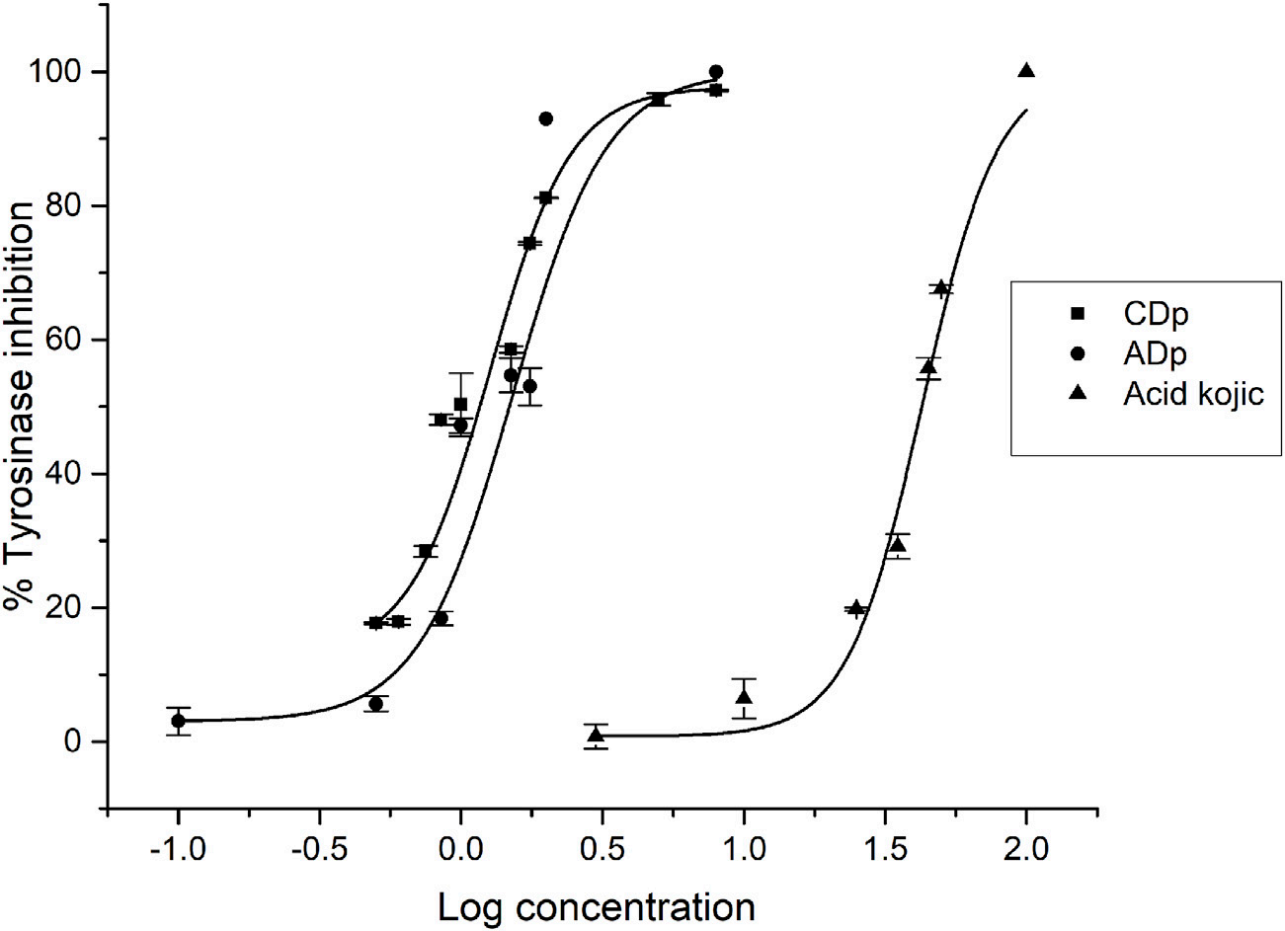
Curve of concentration versus inhibition response of mushroom tyrosinase activity by CDp, ADp, and kojic acid (positive control). CDp: *D. pazensis* chloroform; ADp: *D. pazensis* ethyl acetate. Data points represent mean ± standard error of mean (SEM) from independent replicates (*n* = 3). The concentration-response curves were fitted using a [Dose response fit], and significant differences were not observed between treatments (*p* < 0.05).

Each *D. pazensis* root extract was tested at different concentrations. Extracts that showed more than 50% inhibition at 2 μg/mL (Figure 2) were used to estimate their IC_50_ values through nonlinear fitting of the concentration-response data. Kojic acid served as the positive control. The CDp exhibited an IC_50_ value of 1.27 ± 0.12 μg/mL, followed by the ADp with an IC_50_ of 1.51 ± 0.02 μg/mL, which do not show significantly different activity levels (*p* < 0.05). The IC_50_ of the positive control (kojic acid) was 44.58 ± 0.76 μg/mL (Figure 3).

Recently, we reported the tyrosinase inhibitory activity of other ADp obtained from *D. elegans*, *D. boliviana,* and *D. leporina* (Peralta et al., 2025). The results revealed an interesting inhibitory activity performed by *D. elegans* extract, with comparable biological activity to ADp, the last evaluated in the present study. Unlike the other *Dalea* species, only the chloroformic extract of *D. pazensis* exhibited tyrosinase activity inhibition.

### 3.5 Antifungal activity of *D. pazensis* against *C. albicans*

**FIGURE 4:**
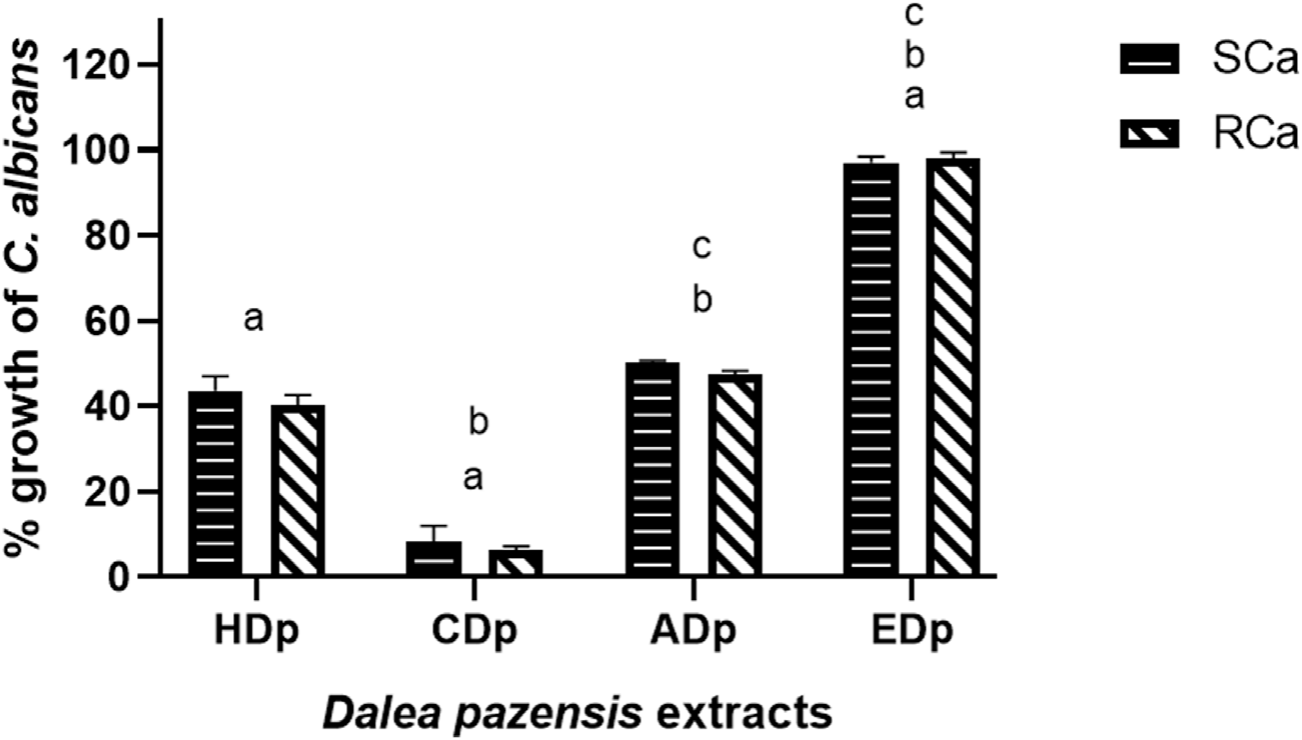
Antifungal activity of the hexane (HDp), chloroform (CDp), ethyl acetate (ADp), and ethanolic (EDp) extracts of *D. pazensis*, against *C. albicans strains.* ^a^ *p* < 0.0001, significant difference between HDp vs. CDp and EDp at 250 μg/mL for the SCa and RCa strains. ^b^ *p* < 0.0001, significant difference between CDp vs. ADp and EDp at 250 μg/mL for the SCa and RCa strains. ^c^ *p* < 0.0001, significant difference between ADp vs. EDp at 250 μg/mL for the SCa and RCa strains.

**FIGURE 5:**
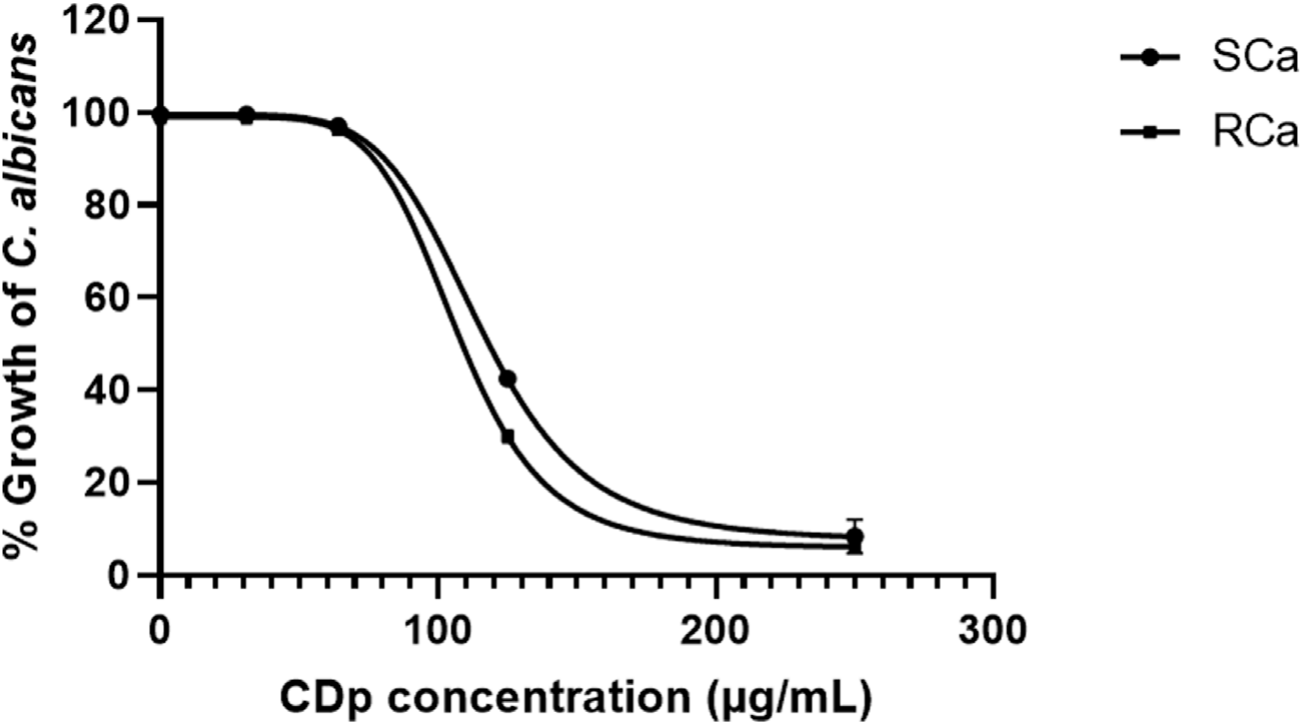
Inhibition of *C. albicans* SCa and RCa strains by the chloroform extract (CDp) of *D. pazensis*. Dose-dependent growth inhibition was observed, with the MIC determined at 125 μg/mL and over 90% inhibition achieved at 250 μg/mL.

The antifungal activity of different *D. pazensis* extracts and the EO was assessed against 2  *C. albicans* strains: an azole-sensitive (SCa) and an azole-resistant (RCa) clinical isolate. The results of the antifungal effect of the *e*xtracts and EO from *D. pazensis* against *C. albicans* strains are summarized in Table 2. The CDp extract showed the strongest antifungal effect, with a minimum inhibitory concentration (MIC) of 125 μg/mL. At this concentration, CDp inhibited SCa and RCa growth by 58.22% and 69.65%, respectively. At 250 μg/mL, the same extract inhibited both strains by over 90% (Figures 4, 5), confirming its strong, dose-dependent fungistatic activity. In contrast, the HDp and ADp showed moderate antifungal activity, with MIC values of 250 μg/mL. At this concentration, the HDp extract inhibited fungal growth by about 70%. In comparison, the ADp reached 50% inhibition for both SCa and RCa strains (Figure 4). The EDp did not exhibit notable antifungal activity at concentrations up to 500 μg/mL and was therefore excluded from further analysis.

**TABLE 2:** Minimum inhibitory concentration values (μg/mL) of organic extracts: hexane (HDp), chloroform (CDp), ethyl acetate (ADp), ethanolic (EDp), and essential oil (EO) of *Dalea pazensis* against sensitive (SCa) and resistant (RCa) *Candida albicans* strains. Positive control, fluconazole (FLZ).

*Candida* albicans strain	MIC values (μg/mL)					
	HDp	CDp	ADp	EDp	EO	FLZ
SCa	250	125	250	>1,000	500	4
RCa	250	125	250	>1,000	500	32

The antifungal activity observed in *D. pazensis* extracts is comparable to, or even exceeds, that reported in our previous study involving *D. boliviana*, *D. elegans*, and *D. leporina* (Peralta et al., 2025). In this study, the chloroform root extract of *D. pazensis* showed strong inhibition against both azole-sensitive and azole-resistant *C. albicans* strains, with MIC values as low as 125 μg/mL and over 90% growth inhibition at 250 μg/mL. Conversely, although we previously demonstrated that all species had antifungal properties, only *D. boliviana* extracts showed similar potency at comparable concentrations. This suggests that *D. pazensis*, like *D. boliviana*, is a promising source of antifungal agents within the genus. The high relative abundance of compound 1 in *D. pazensis* may, once again, support this bioactivity, reinforcing its proposed role as a key antifungal metabolite in *Dalea* species.

**FIGURE 6:**
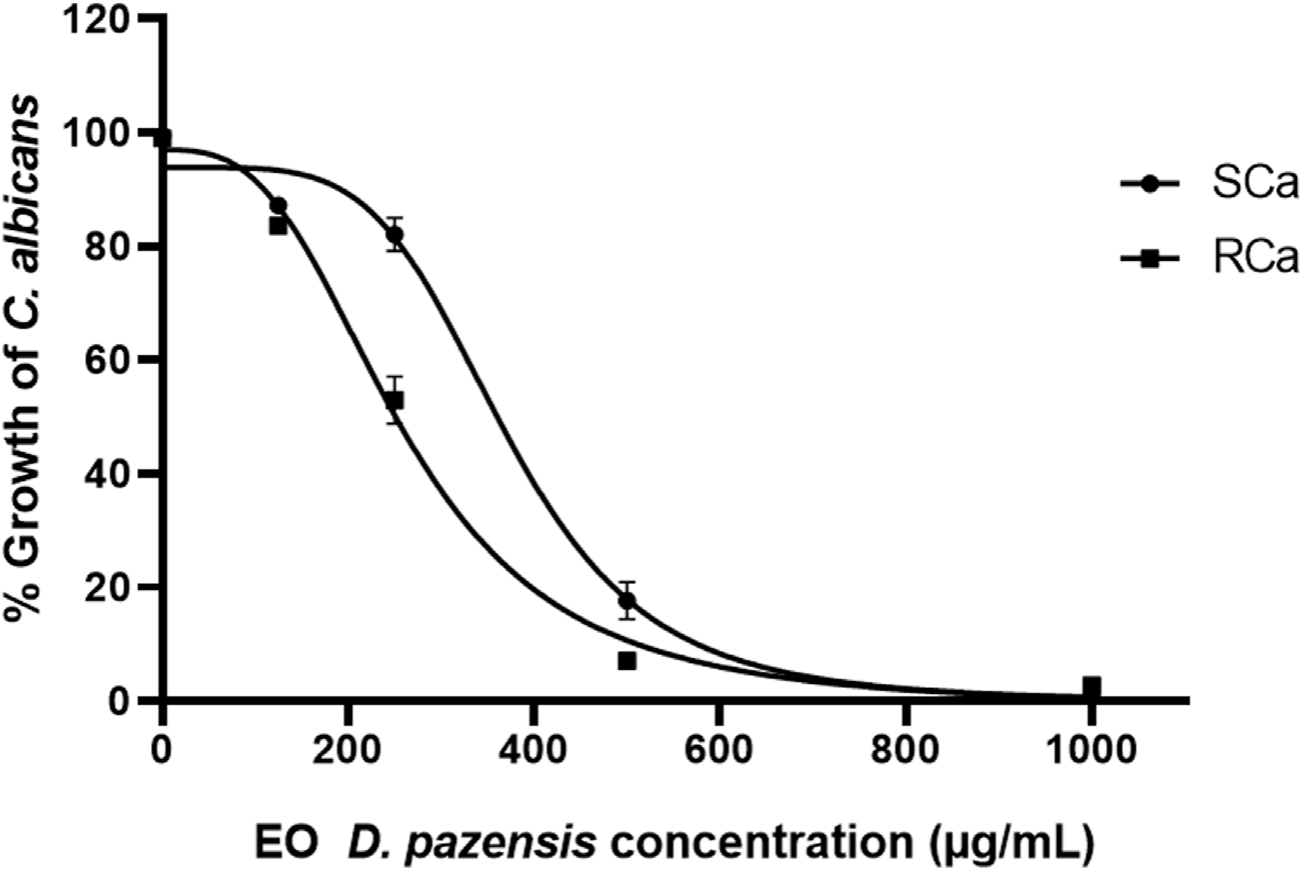
Antifungal activity of the essential oil (EO) from *D. pazensis* aerial parts against *C. albicans* strains. The minimum inhibitory concentration (MIC) was determined to be 500 μg/mL for both the azole-sensitive strain (SCa) and the azole-resistant strain (RCa), based on absorbance measurements at 540 nm.

The EO from the aerial parts of *D. pazensis* also showed antifungal effects, with an MIC of 500 μg/mL against both strains, as measured spectrophotometrically at 540 nm (Figure 6). For comparison, the reference antifungal, fluconazole, had MICs of 8 μg/mL for SCa and 128 μg/mL for RCa under the same test conditions.

Based on the results, we conducted a targeted chemical characterization of the most bioactive extracts from *D. pazensis* roots. The analyses employed advanced techniques, including UPLC-MS/MS, to identify and characterize the relevant compounds. The relative abundance of each identified compound in the extracts was reported. Additionally, the EO was analyzed by GC-MS.

### 3.6 Flavanone profiling in active *Dalea pazensis* extracts using UPLC-MS/MS

Based on the results of the anti-tyrosinase and antifungal assays, it was decided to determine the chemical composition of *D. pazensis* by identifying *Dalea* compounds 1-6 and their relative abundances in CDp and ADp extracts using UPLC-MS/MS in MRM mode.

Flavanones 1, 2, and 5 were identified in the CDp extract of *D. pazensis*, with 1 showing the highest relative abundance (area: 6240887.00, Supplementary Table S2; Supplementary Figure S1). The ADp extract contained most of the evaluated flavonoids, except for compound 4. Again, compound 1 has the highest relative abundance in the extract (area: 1843719.25, Supplementary Table S2; Supplementary Figure S1). Therefore, based on the chemical analysis, compound 1 is present as the majority compound of both extracts. Recently, we reported its presence and relative abundance in different extracts from *Dalea* species: *D. boliviana*, *D. elegans*, and *D. leporina*. Additionally, its presence has been noted in other *Dalea* species: *Dalea scandens* var. p*aucifolia* (J.M. Coult.). Barneby, *Dalea searlsiae* (A. Gray) Barneby, *Dalea ornata* (Hook.) Eaton & J. Wright, *Dalea versicolor* var. *sessilis* (A. Gray) Barneby, and *Dalea pogonathera* A. Gray. Its presence across several *Dalea* species supports, at least in part, the idea that 1 is an important marker compound in this genus.

Regarding the observed tyrosinase activity, both extracts demonstrated notable tyrosinase inhibitory activity, which could be attributed to the presence of compound 1. Another compound that could collaborate with this activity is compound 5. Both compounds have demonstrated anti-tyrosinase activity, so the activity observed in both extracts could be due to a synergistic effect arising from the presence of different prenylflavanones (1 and 5) belonging to the *Dalea* genus. More studies are necessary in order to corroborate this hypothesis.

Given the qualitative composition of the CDp extract compared to ACp, we propose quantitatively extracting 1, a known anti-tyrosinase compound from CDp (Peralta et al., 2014).

Our current findings on the anti-tyrosinase and antifungal activities of *D. pazensis* root extracts align with our previous work on other *Dalea* species (Peralta et al., 2025), where we reported distinct biological profiles for *D. boliviana*, *D. elegans*, and *D. leporina*, highlighting the importance of specific prenylated flavanones. In this study, both the CDp and ADp extracts of *D. pazensis* showed significant inhibitory activity against *C. albicans* and tyrosinase, with compound 1 identified as the main flavanone in both extracts. Consistent with our earlier results, this compound remains a key bioactive metabolite across several *Dalea* species, supporting its role as a potential chemotaxonomic marker and an important contributor to the genus’s pharmacological potential.

The CDp of *D. pazensis* exhibited the most potent antifungal activity among the tested root extracts. This bioactivity is consistent with its phytochemical composition, particularly the high abundance of compound 1, which our group previously reported to exert significant antifungal effects against azole-resistant C. albicans (RCa) by inhibiting MDR mechanisms, including efflux pump overexpression (Peralta et al., 2012). As the main component of CDp, compound 1 is likely to be key to the observed antifungal effect.

In addition to compound 1, CDp also contains compound 5, the second-most abundant flavanone. This compound, originally isolated from *D. boliviana*, demonstrated antifungal activity against the same MDR strain (Negro et al., 2024). The presence of both bioactive compounds in significant amounts provides a strong chemical basis for CDp’s potent antifungal activity.

The ADp also showed antifungal activity against RCa, although its effectiveness was lower than that of CDp. UPLC-MS/MS analysis identified several prenylated flavanones in ADp, including compounds 1, 4, 5, and 6, all of which have been previously characterized by our group and linked to antifungal activity against resistant *C. albicans* strains (Peralta et al., 2012; Peralta et al., 2015; Peralta et al., 2019; Negro et al., 2024).

The antifungal profiles observed for CDp and ADp suggest that the activity cannot be attributed solely to the most abundant compound, but rather to the combined action of multiple prenylated flavanones. Prenylation increases lipophilicity and membrane affinity, which may facilitate cooperative interactions among these metabolites at the fungal cell envelope. Previous studies in *Dalea* species (Peralta et al., 2012; Negro et al., 2024) have shown that structurally related flavanones display complementary mechanisms, including efflux pump inhibition and interference with oxidative homeostasis. The co-occurrence of compounds 1, 5, and other prenylated derivatives in both extracts may therefore enhance antifungal efficacy through additive or synergistic effects, particularly in MDR *C. albicans*, where simultaneous modulation of membrane integrity and transport systems is essential to overcome resistance.

Our previous studies on root extracts from other *Dalea* species have shown similar patterns, with chloroform extracts—especially those from *D. boliviana*—exhibiting the strongest antifungal activity (Peralta et al., 2025). These consistent results further highlight the importance of chloroform-soluble fractions in the *Dalea* genus as a valuable source of prenylated flavanones with notable antifungal potential, especially against multidrug-resistant (MDR) *C. albicans*. Therefore, CDp stands out as a key extract in the search for plant-based antifungal agents capable of overcoming resistance mechanisms in pathogenic fungi.

Taken together, the chemical profiles of CDp and ADp provide a clear rationale for their biological activities. The higher antifungal potency of CDp correlates with its enriched levels of compound 1, a prenylated flavanone previously demonstrated to inhibit efflux pump–mediated resistance and to disrupt fungal physiology in MDR *C. albicans*. The presence of compound 5, also known for its antifungal effects, further reinforces this activity. Similarly, the strong tyrosinase inhibition observed for both extracts is consistent with the predominance of compound 1, a flavanone with well-documented anti-tyrosinase activity. In ADp, the combined presence of several prenylated flavanones (1, 4, 5, and 6) likely contributes to its intermediate antifungal effect through additive or synergistic interactions. Overall, the qualitative and quantitative flavanone composition provides robust chemical support for the antifungal and enzymatic activities recorded in this study.

### 3.7 Chemical composition of the EO from *D. pazensis*

The chemical analysis of the EO from *D. pazensis* was conducted using GC-MS. The results showed that β-caryophyllene (49.8%) was the main component, followed by lower amounts of other sesquiterpenes (Table 3). β-Caryophyllene is a sesquiterpene well known for its anti-inflammatory, antimicrobial, antioxidant, and cytotoxic properties (Francomano et al., 2019), making its high abundance pharmacologically relevant. This compositional profile differs markedly from that of previously studied Dalea species. In most reported EOs, such as *D. scoparia*, *D. greggii*, *D. lumholtzii*, *D. carthagenensis*, *D. formosa*, *D. foliolosa*, *D. strobilacea*, and *D. mutisii*, major compounds include bisabolol, limonene, α-pinene, and other mono- and sesquiterpenes rather than β-caryophyllene (Lucero et al., 2005; Benites et al., 2016; Villa-Ruano et al., 2017; Muñoz-Acevedo et al., 2019). More recently, the EO of *D. bicolor* was characterized and found to contain bicyclic monoterpenes and oxygenated sesquiterpenes as major constituents, with β-caryophyllene reported at moderate or low levels (Esquivel-Campos et al., 2024). Compared with these species, the EO of *D. pazensis* stands out for its unusually high β-caryophyllene content, further supporting the existence of a distinct chemotype within the genus, characterized by β-caryophyllene as a key compound. This differentiation reinforces the botanical and chemical diversity of *Dalea*, and has important biological implications: β-caryophyllene has documented antifungal activity and membrane-targeting properties (Dahham et al., 2015), which may contribute to the inhibitory effects observed against *C. albicans* in this study.

**TABLE 3:** Chemical composition (%) of the essential oil of aerial parts of *D. pazensis*.

Retention time (min)	RI_exp_ ^a^	RI_lit_ ^b^	Relative content (%)	Identified compound	Chemical formula
6.894	931.3	932	0.80	1*R*-α-Pinene	C_10_H_16_
8.405	976.1	977	1.18	(−)-β-Pinene	C_10_H_16_
20.775	1,384.5	1,385	2.06	α-Copaene	C_15_H_24_
21.970	1,431.0	1,433	41.1	β-Caryophyllene	C_15_H_24_
22.850	1,465.9	1,463	5.42	α-Caryophyllene	C_15_H_24_
25.957	1,594.8	1,596	17.24	Caryophyllene oxide	C_15_H_24_O
26.577	1,622.2	1,613	6.29	Copaborneol	C_15_H_26_O
28.033	1,687.2	1,652	3.34	Eudesmol	C_15_H_26_O

^a^ Kovats index calculated from alkanes series on the MS.

^b^ Kovats index/Retention index from data libraries (NIST) and literature (Babushok et al., 2011).

Taken together, the predominance of β-caryophyllene not only distinguishes the phytochemical identity of *D. pazensis* EO but also strengthens its potential as a natural source of bioactive sesquiterpenes for antifungal development.

Although the prenylated flavanones identified in this study were detected in root extracts, it is reasonable to hypothesize that they interact synergistically with the volatile constituents of the aerial parts, particularly β-caryophyllene. In several *Dalea* species, flavonoids have been detected in both underground and aerial tissues, even though their presence in *D. pazensis* shoots has not yet been investigated. Therefore, a natural co-occurrence of flavanones and EO components within different organs of the plant is plausible. Considering the complementary mechanisms of action, membrane-permeabilizing effects of β-caryophyllene and efflux pump inhibition or membrane disruption associated with prenylated flavanones, the plant may produce metabolite combinations capable of exerting multitarget antifungal activity. Although our experimental design did not evaluate combined extracts, these phytochemical and mechanistic features support the rationale for proposing a synergistic interaction as a working hypothesis for future studies.

## 4 Conclusion

In summary, given that this is the first chemical report of the essential oil and the deepening of prenylflavonoid content in different extracts of *Dalea pazensis*, we consider that this study highlights their pharmacological potential, especially through its CDp, which showed the strongest antifungal and tyrosinase inhibitory activities among all tested samples. Based on its qualitative composition, CDp is proposed as a valuable source for the quantitative isolation of (2*S*)-5,7,2′,4′-tetrahydroxy-5′-(1‴,1‴-dimethylallyl)-8-prenylflavanone (compound 1 or 8PP), a prenylated flavanone known for its dual antifungal and anti-tyrosinase effects (Peralta et al., 2014; Peralta et al., 2015). Additionally, 8PP has been reported to have neuroprotective effects (Santi et al., 2024), further emphasizing its importance as a multifunctional lead compound.

The GC-MS analysis of the essential oil from the aerial parts revealed β-caryophyllene as the main constituent (41.1%), indicating a distinct chemotype within *D. pazensis* compared to other species in the genus. This unique volatile profile, along with the presence of bioactive prenylflavanones, highlights the distinctive phytochemical profile and pharmacological significance of this species.

Collectively, the results support *D. pazensis* as a promising native source of multifunctional natural products with potential applications in antifungal, dermatological, and neuroprotective therapies, and lay the groundwork for future bioguided isolation and formulation studies.

## Data Availability

The original contributions presented in the study are included in the article/Supplementary Material, further inquiries can be directed to the corresponding authors.

## References

[B1] Babushok V. I. Linstrom P. J. Zenkevich I. G. (2011). Retention indices for frequently reported compounds of plant essential oils. J. Phys. Chem. Ref. Data 40, 043101. 10.1063/1.3653552

[B2] Bedoya Aguirre E. N. Santi M. D. Negro M. F. Echeverría J. Paulino Zunini M. Peralta M. A. (2025). Chromene flavanones from Dalea boliviana as xanthine oxidase inhibitors: *in vitro* biological evaluation and molecular docking studies. Front. Pharmacol. 16, 1576390. 10.3389/fphar.2025.1576390 40351436 PMC12062022

[B3] Benites J. Moiteiro C. Figueiredo A. C. Rijo P. Buc-Calderon P. Bravo F. (2016). Chemical composition and antimicrobial activity of essential oil of peruvian Dalea strobilacea Barneby. Bol. Latinoam. del Caribe Plantas Aromáticas 15, 429–435.

[B4] CLSI (2017). M27-A4: reference method for broth dilution antifungal susceptibility testing of yeasts. Available online at: https://clsi.org/standards/products/microbiology/documents/m27/.

[B5] Dahham S. Tabana Y. Iqbal M. Ahamed M. Ezzat M. Majid A. (2015). The anticancer, antioxidant and antimicrobial properties of the sesquiterpene β-Caryophyllene from the essential oil of Aquilaria crassna. Molecules 20, 11808–11829. 10.3390/molecules200711808 26132906 PMC6331975

[B6] Densmore F. (2005). Strength of the earth: the classic guide to Ojibwe uses of native plants. St. Paul, MN: Minnesota Historical Society.

[B7] Esquivel-Campos A. L. Sánchez-Pérez L. González-Chávez M. M. Reyes-Ponce A. Zapata-Flores E. de J. Pérez-Gutiérrez S. (2024). Antimicrobial and antioxidant activities of four essential oils. J. Mex. Chem. Soc. 68, 593–608. 10.29356/jmcs.v68i4.2309

[B8] Francomano F. Caruso A. Barbarossa A. Fazio A. La Torre C. Ceramella J. (2019). β-Caryophyllene: a sesquiterpene with countless biological properties. Appl. Sci. 9, 5420. 10.3390/app9245420

[B9] Gaudio M. D. Santi M. D. Cabrera J. L. Peralta M. A. Ortega M. G. (2020). Dalea extracts as potential for phyto-ingredients: antioxidant, antityrosinase, antifungal and cytotoxicity *in vitro* evaluations. Ciências Biológicas Interface com vários Saberes 2, 130–143. 10.22533/at.ed.38220021012

[B10] Gilardoni G. Montalván M. Ortiz M. Vinueza D. Montesinos J. V. (2020). The flower essential oil of Dalea mutisii kunth (Fabaceae) from Ecuador: chemical, enantioselective, and olfactometric analyses. Plants 9, 1403. 10.3390/plants9101403 33096831 PMC7589571

[B11] Heinrich M. Jalil B. Abdel-Tawab M. Echeverria J. Kulić Ž. McGaw L. J. (2022). Best practice in the chemical characterisation of extracts used in pharmacological and toxicological research—The ConPhyMP—Guidelines12. Front. Pharmacol. 13, 953205. 10.3389/fphar.2022.953205 36176427 PMC9514875

[B12] Lucero M. E. Estell R. E. Sedillo R. L. (2005). The composition of Dalea formosa oil determined by steam distillation and solid-phase microextraction. J. Essent. Oil Res. 17, 645–647. 10.1080/10412905.2005.9699022

[B13] McCaughey W. F. Buehrer T. F. (1961). Essential oil plants of Southern Arizona. J. Pharm. Sci. 50, 658–660. 10.1002/jps.2600500807 13773779

[B14] Muñoz-Acevedo A. González M. del C. Stashenko E. E. (2019). Volatile fractions and essential oils of the leaves and branches of Dalea carthagenensis (Jacq.) J.F. macbr. from northern region of Colombia. J. Essent. Oil Bear. Plants 22, 774–788. 10.1080/0972060X.2019.1623720

[B15] Negro M. F. Ortega M. G. Peralta M. A. (2024). Bioprospecting prenyl flavanones from Dalea boliviana: structural insights and antifungal properties against azole-resistant Candida albicans. Rev. Bras. Farmacogn. 34, 785–792. 10.1007/s43450-024-00526-7

[B16] Peralta M. A. Ortega M. G. Agnese A. M. Cabrera J. L. (2011). Prenylated flavanones with anti-tyrosinase activity from Dalea boliviana. J. Nat. Prod. 74, 158–162. 10.1021/np1004664 21226489

[B17] Peralta M. Calise M. Fornari M. Ortega M. Diez R. Cabrera J. (2012). A prenylated flavanone from Dalea elegans inhibits rhodamine 6 G efflux and reverses fluconazole-resistance in Candida albicans. Planta Med. 78, 981–987. 10.1055/s-0031-1298627 22673834

[B18] Peralta M. A. Santi M. D. Agnese A. M. Cabrera J. L. Ortega M. G. (2014). Flavanoids from Dalea elegans: chemical reassignment and determination of kinetics parameters related to their anti-tyrosinase activity. Phytochem. Lett. 10, 260–267. 10.1016/j.phytol.2014.10.012

[B19] Peralta M. A. da Silva M. A. Ortega M. G. Cabrera J. L. Paraje M. G. (2015). Antifungal activity of a prenylated flavonoid from Dalea elegans against Candida albicans biofilms. Phytomedicine 22, 975–980. 10.1016/j.phymed.2015.07.003 26407939

[B20] Peralta M. A. Santi M. D. Cabrera J. L. Ortega M. G. (2019). “Dalea genus, chemistry, and bioactivity studies,” in Studies in natural products chemistry (Elsevier), 307–341. 10.1016/B978-0-444-64185-4.00008-3

[B21] Peralta M. A. Negro M. F. Aguirre E. N. B. Santi M. D. Ortega M. G. (2025). Antifungal and anti-tyrosinase activities of dalea species extracts: differential biological effects and their correlation with phytochemical content *via* UPLC-MS/MS profiling. Rev. Bras. Farmacogn. 35, 567–574. 10.1007/s43450-025-00641-z

[B22] Santi M. D. Peralta M. A. Mendoza C. S. Cabrera J. L. Ortega M. G. (2017). Chemical and bioactivity of flavanones obtained from roots of Dalea pazensis rusby. Bioorg. Med. Chem. Lett. 27, 1789–1794. 10.1016/j.bmcl.2017.02.058 28268138

[B23] Santi M. D. Carvalho D. Dapueto R. Bentura M. Zeni M. Martínez-González L. (2024). Prenylated flavanone isolated from Dalea species as a potential multitarget-neuroprotector in an *in vitro* Alzheimer’s disease mice model. Neurotox. Res. 42, 23. 10.1007/s12640-024-00703-5 38578482

[B24] Villa-Ruano N. Pacheco-Hernandez Y. Rubio-Rosas E. Zarate-Reyes J. A. Lozoya-Gloria E. Cruz-Duran R. (2017). Chemical profile, nutraceutical and anti-phytobacterial properties of the essential oil from Dalea foliolosa (Fabaceae). Emir. J. Food Agric. 29, 724–729. 10.9755/ejfa.2017.v29.i9.99

[B25] White T. C. Holleman S. Dy F. Mirels L. F. Stevens D. A. (2002). Resistance mechanisms in clinical isolates of Candida albicans. Antimicrob. Agents Chemother. 46, 1704–1713. 10.1128/AAC.46.6.1704-1713.2002 12019079 PMC127245

