# Supplementary material for: Phytochemical characterization and pharmacological evaluation of aerial and root parts of Dalea pazensis Rusby [Fabaceae]
Source: Front Pharmacol. 2026 Jan 5;16:1717359. doi: 10.3389/fphar.2025.1717359 (PMC12812976; doi:10.3389/fphar.2025.1717359)
Supplement: Supplementary file 1 [file Supplementaryfile1.docx]

**Table S1.** Different MRM parameters (precursor, several product ions, collision energy, cone voltage, and retention times) of flavonoids **1**-**6** obtained from *Dalea* species.

| **Flavonoid** | **Retention time (min)** | **Precursor ion (Da)** | **Quantifier ion (a)**  **(Da)** | **Qualifier**  **Ion (b)**  **(Da)** | **Qualifier**  **Ion (c)**  **(Da)** | **Collision Energy**  **(eV)** | | | **Cone voltage**  **(V)** |
| --- | --- | --- | --- | --- | --- | --- | --- | --- | --- |
|  |  |  |  |  |  | **a** | **b** | **c** |  |
| **1** | 1.02 | 423 | 193 | 228.9 | 124.2 | 16 | 16 | 28 | 62 |
| **2** | 1.30 | 323 | 163.9 | 133 | 218.9 | 20 | 28 | 22 | 60 |
| **3** | 0.85 | 283 | 163.9 | 135.9 | 107.9 | 20 | 28 | 30 | 54 |
| **4** | 1.78 | 407.1 | 193 | 124.2 | 132 | 16 | 36 | 40 | 26 |
| **5** | 1.77 | 407.1 | 193 | 124.2 | 149.1 | 16 | 36 | 32 | 62 |
| **6** | 1.35 | 336.9 | 191 | 146.9 | 118.9 | 14 | 24 | 34 | 48 |

**Table S2.** UPLC-MS/MS analysis of bioactive compounds in chloroform (CDp) and ethyl acetate (ADp) extracts of *Dalea pazensis*

| **Flavonoid** | **CDp** | | | **ADp** | | |
| --- | --- | --- | --- | --- | --- | --- |
|  | **Retention time (min)** | **Transition (*m/z*)** | **Area** | **Retention time (min)** | **Transition (*m/z*)** | **Area** |
| **1** | 0.95 | 423 > 193 | 6240887.00 | 0.96 | 423 > 193 | 976246.56 |
|  | 0.96 | 423 > 228.9 |  | 0.97 | 423 > 228.9 |  |
| **2** | 1.22 | 323 > 163.9 | 1239.40 | 1.22 | 323 > 163.9 | 1162.40 |
|  | 1.22 | 323 > 133 |  | 1.22 | 323 > 133 |  |
| **3** | - | - | - | - | - | - |
|  | - | - |  | - | - |  |
| **4** | - | - | - | 1.78 | 407.1>193 | 45301.71 |
|  | - | - |  | 1.78 | 407.1>124.2 |  |
|  | - | - |  | 1.78 | 407.1>132 |  |
| **5** | 1.80 | 407.1 > 193 | 13720.46 | 1.78 | 407.1>193 | 53532.57 |
|  | 1.80 | 407.1 > 124.2 |  | 1.78 | 407.1>124.2 |  |
|  | 1.80 | 407.1>149.1 |  | 1.78 | 407.1>149.1 |  |
| **6** | - | - | - | 1.26 | 336.9 > 191 | 3348.30 |
|  | - | - |  | 1.26 | 336.9 > 146.9 |  |

**
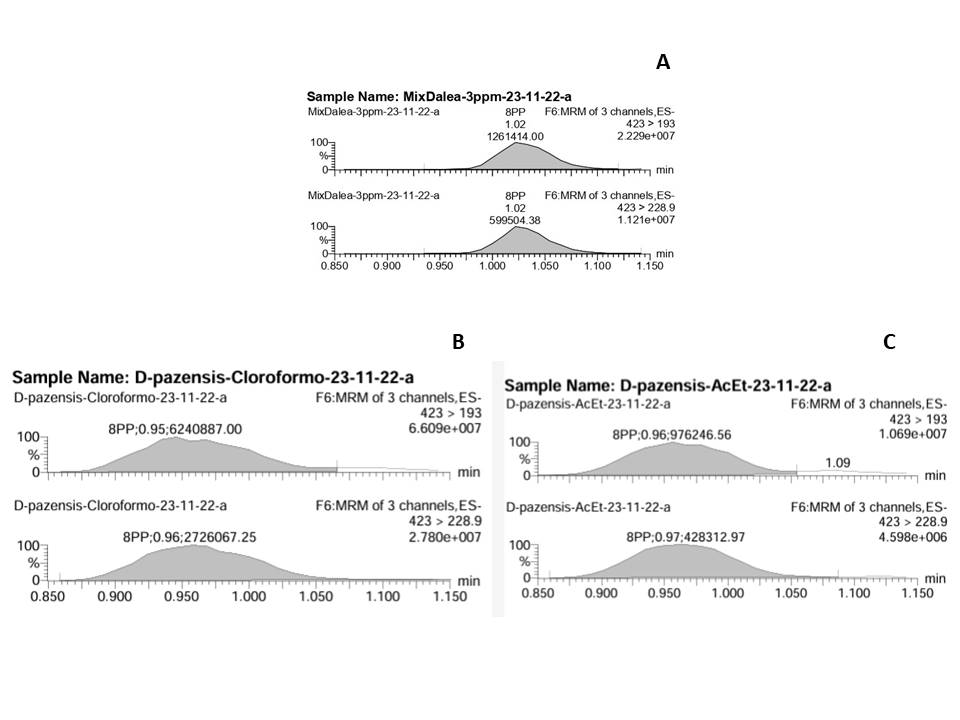
**

**Figure S1.** Representative MRM of 1 in the chloroform and ethyl acetate extracts of *Dalea pazensis:* A: MRM of **1,** B: MRM of **1** in CDp, C: MRM of **1** in ADp.


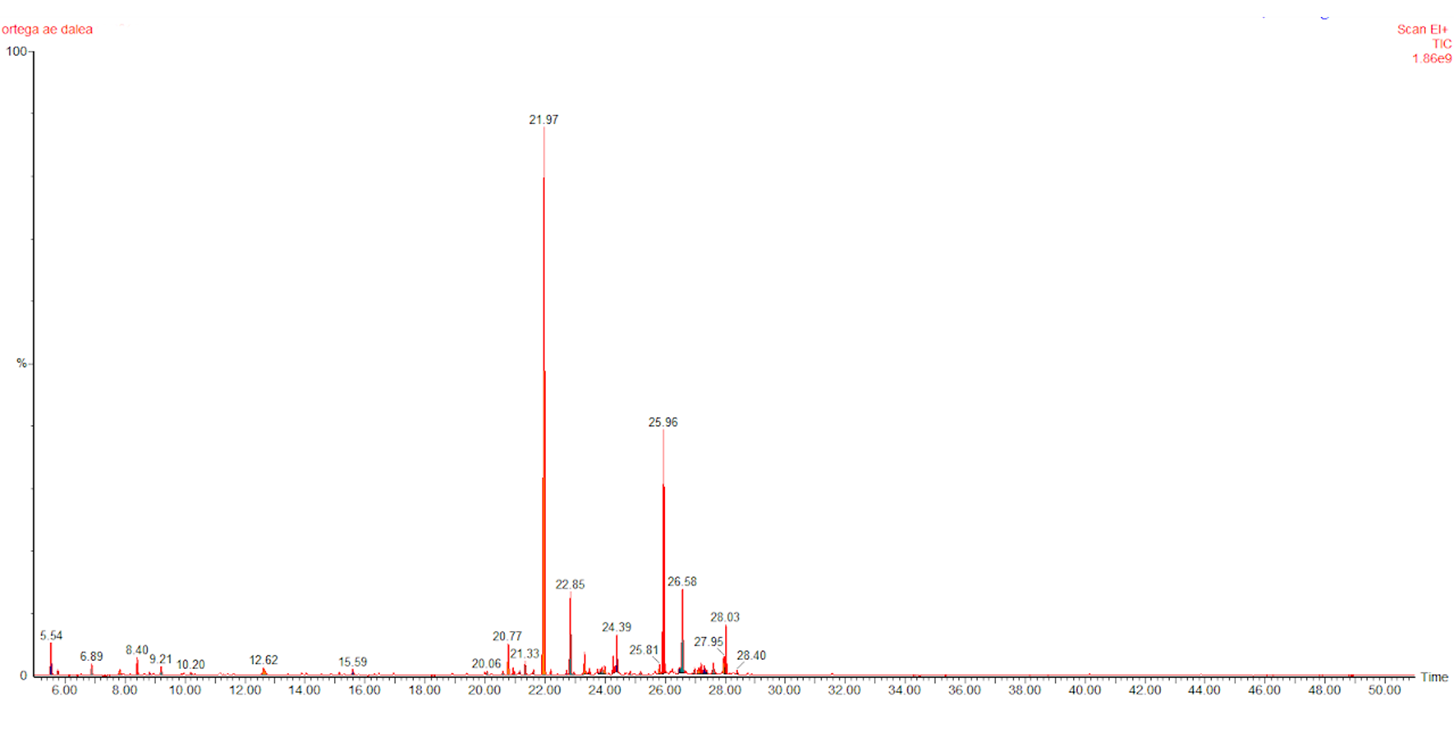


**Figure S2.** GC/MS chromatogram of the essential oil of *Dalea pazensis.*
